# Latent profile analysis of self-efficacy in information security and positive coping level among nursing students: a multicenter cross-sectional study in China

**DOI:** 10.3389/fpsyg.2026.1758669

**Published:** 2026-03-23

**Authors:** Yingxue Niu, Shiming Chen, Min Hu, Yifang Li

**Affiliations:** 1 Department of Nursing, Anhui University of Chinese Medicine, Hefei, China; 2 Laboratory of Geriatric Nursing and Health, Anhui University of Chinese Medicine, Hefei, China

**Keywords:** factors, latent profile analysis, nursing students, positive coping, self-efficacy in information security

## Abstract

**Objective:**

This study aimed to (1) identify latent profiles of nursing students based on self-efficacy in information security and positive coping, and (2) examine factors associated with self-efficacy in information security.

**Methods:**

A cross-sectional study was conducted on nursing students. Latent profile analysis was used to identify potential profiles based on self-efficacy in information security and positive coping levels. Subsequently, multivariate linear regression was conducted to identify factors associated with the self-efficacy in information security.

**Results:**

A total of 1,832 nursing students participated in this study. Four distinct latent profiles were identified: low self-efficacy-negative coping profile (24.0%), low self-efficacy-positive coping profile (10.6%), awareness-dominant profile (56.2%), and high self-efficacy-positive coping profile (9.2%). Multivariate linear regression analysis further revealed that education level, grade, clinical internship experience, completion of a nursing informatics course, receipt of information security training, and experience of digital victimization were associated with self-efficacy in information security (*P* < 0.05).

**Conclusion:**

The findings revealed heterogeneous profiles and identified factors associated with self-efficacy in information security among nursing students. These results underscore the importance of developing tailored interventions for this population.

## 1 Introduction

The rapid integration of digital technologies into nursing education has reshaped students learning, communication, and information access. While these innovations offer notable benefits, their growing use raises information security concerns, including data breaches, privacy violations, and the misuse of sensitive personal data (Allen et al., 2025; Wei et al., 2025). Global and national policies emphasize the integration of digital literacy and cybersecurity into health professional training. *The World Health Organization's Global Strategy on Digital Health* 2020–*2025* calls for the integration of digital health into the education and training of healthcare professionals and highlights cybersecurity as an essential component of resilient health systems ( World Health Organization, 2021). In China, the *Healthy China 2030 blueprint* advocates for enhanced public awareness and competence in information protection (China, 2016). Although policy initiatives have emphasized the importance of cybersecurity competencies, translating these macro-level expectations into nursing students' consistent and autonomous digital security behaviors remains challenging. A key factor in this translation is the development of students' self-efficacy in information security (SEIS). As a core psychological mechanism linking knowledge to practice, the SEIS plays a critical role in shaping actual behavioral engagement.

Rooted in Bandura's social cognitive theory (Bandura, 1986), the SEIS is defined as the perceived ability to safeguard digital systems and prevent information security violations, which enables individuals to implement appropriate protective measures and adhere to responsible data practices (Rhee et al., 2009). Previous research in healthcare contexts has indicated that individuals with higher SEIS exhibit greater risk awareness, stronger engagement in protective behaviors, and increased motivation to prevent security breaches (Sari et al., 2022). It has emerged as a crucial psychological and behavioral resource for students operating in increasingly digital environments. A higher SEIS is associated with increased vigilance, a stronger sense of ethical responsibility, and more proactive engagement in secure digital practices (Xue et al., 2023). For nursing students who routinely handle sensitive patient information, use electronic health records, and engage with online learning platforms, strong SEIS supports secure data practices and strengthens their professional digital identity and resilience to online threats (Amin et al., 2025; Reierson et al., 2025; Yang and Jang, 2025).

Given that digital threats often function as psychological stressors, students' responses to these challenges are pivotal in understanding their subsequent behavioral adjustments. In this context, positive coping, characterized by proactive problem-solving, constructive emotional regulation, and adaptive help-seeking, emerges as a key mechanism through which individuals mitigate stress and maintain wellbeing (Aldbyani et al., 2025; Wu et al., 2020). Previous research suggests that individuals who employ positive coping strategies experience lower stress levels and better psychological adaptation when confronted with academic or digital challenges (Rodrigues et al., 2023; Shiraly et al., 2024). Therefore, examining positive coping levels is essential for understanding how students translate the SEIS into resilient and adaptive outcomes.

Despite the growing interest in self-efficacy in nursing education, research on the SEIS among nursing students remains limited. Crucially, treating students as a homogenous group by focusing only on variable-centered associations risks overlooking distinct subgroups with unique needs and challenges. Most existing studies conceptualize SEIS as a single, uniform construct, ignoring potential heterogeneity among individuals (Warshawski et al., 2019; Xue et al., 2023). Integrating positive coping levels into this profiling offers a more comprehensive view of both cognitive confidence and behavioral strategies. To address these gaps, this study adopted a person-centered approach to identify distinct profiles based on nursing students' SEIS and positive coping levels. Using Latent Profile Analysis (LPA), we aimed to identify profiles characterized by distinct configurations of cognitive confidence and behavioral coping tendencies, thereby extending existing variable-centered findings that treat students as a homogeneous population. These insights offer an empirical basis for developing targeted, profile-specific educational strategies that strengthen cybersecurity competence and promote psychological resilience in increasingly digitalized nursing learning environments.

## 2 Methods

### 2.1 Design and data collection

A cross-sectional survey was distributed using the Wenjuanxing platform (https://www.wjx.cn/, a popular online survey platform in China). Participants were presented with a standardized introduction explaining the study's purpose, nature, and completion instructions. Participation was voluntary and anonymous. To ensure data quality, the platform limited each IP address to a single response and required all questions to be completed before submission. Responses with patterned answers, defined as selecting the same response option for all items across the scale, or with completion times of less than 3 min were excluded.

### 2.2 Participants

From April to July 2025, convenience sampling was employed to recruit nursing students from three vocational schools, colleges and universities in East China. The inclusion criteria were: (a) full-time nursing students; (b) provision of informed consent and voluntary participation. The exclusion criteria were: (a) students on academic leave or suspension; (b) students with a diagnosed mental health disorder.

### 2.3 Measures

#### 2.3.1 General information questionnaire

This questionnaire, developed by the research team following a comprehensive literature review, was used to collect demographic information, including gender, age, education level, grade, monthly per capita household income, clinical internship experience, and other relevant characteristics.

#### 2.3.2 Self-efficacy in information security scale (SEIS)

The Self-Efficacy in Information Security Scale (SEIS) is a 19-item instrument originally developed in Chinese by Nie R (Nie, 2021). The scale comprises four dimensions: (a) judgment skills regarding personal information security, (b) control over content on mobile social platforms, (c) perceptions of one's information security status, and (d) protective behaviors for personal information. All items were rated on a 5-point Likert scale from 1 (strongly disagree) to 5 (strongly agree), with total scores ranging from 19 to 95. Higher scores indicate a stronger SEIS. To further examine construct validity in the present sample, confirmatory factor analysis (CFA) was conducted. The four-factor model demonstrated excellent model fit: χ^2^ (146) = 453.77, *P* < .001; Robust CFI = 0.984; Robust TLI = 0.982; Robust RMSEA = 0.039 (90% CI: 0.035–0.043); SRMR = 0.024. All standardized factor loadings were statistically significant (*P* < .001) and ranged from 0.653 to 0.909, supporting the construct validity of the instrument in this population. The Cronbach's alpha coefficient in this study was 0.951.

#### 2.3.3 Simplified coping style questionnaire (SCSQ)

The SCSQ, developed and revised by Chinese scholar Xie YN (Xie, 1998), comprises 20 items across two dimensions: positive coping (items 1–12) and negative coping (items 13–20). Each item is rated on a 4-point scale ranging from 0 (not at all) to 3 (frequently). Only the positive coping subscale was used in this study, with total scores ranging from 0 to 36. Higher scores reflect a stronger tendency to adopt positive coping strategies. Construct validity was evaluated using CFA for the positive coping dimension. The CFA demonstrated excellent model fit: χ^2^ (54) = 62.95, *P* = 0.189; Robust CFI = 0.999; Robust TLI = 0.999; Robust RMSEA = 0.011 (90% CI: 0.000–0.020); SRMR = 0.010. All standardized factor loadings were statistically significant (*P* < .001) and ranged from 0.670 to 0.854, supporting the construct validity of the scale in this sample. The Cronbach's alpha coefficient in this study was 0.946.

### 2.4 Data analysis

Data were managed using Microsoft Excel 2019, and all statistical analyses were conducted using R 4.3.3 and Mplus 8.3. LPA was performed in Mplus 8.3 using standardized scores (z-scores) of the four SEIS dimensions and positive coping, ensuring that all indicators were equally weighted. Model fit was evaluated using the Akaike information criterion (AIC), Bayesian information criterion (BIC), sample size-adjusted BIC (aBIC), Lo-Mendell-Rubin likelihood ratio test (LMR-LRT), bootstrap likelihood ratio test (BLRT), and entropy. Lower AIC, BIC, and aBIC indicate a better model fit. A significant *P*-value (< 0.05) for LMR-LRT and BLRT indicates that the k-profile model provides a significantly better fit than the (k-1)-profile model. Entropy values closer to 1.0 indicate a higher classification accuracy.

Multiple linear regression analyses were conducted using R4.3.3 to examine the factors associated with SEIS. Multicollinearity was assessed using variance inflation factors (VIF) calculated using the car package. Residual normality was evaluated using Q–Q plots and by calculating skewness and kurtosis using the moments package. Homoscedasticity was examined using the Breusch–Pagan test, implemented in the lmtest package. Given the evidence of heteroscedasticity, heteroscedasticity-consistent (HC3) robust standard errors were applied using the sandwich and lmtest packages. The Standardized regression coefficients were obtained using the lm.beta package. Data import was performed using the rio package.

## 3 Results

### 3.1 Participants' characteristics

Among the 1,832 participants, the majority were female (81.9%), and the mean age was 18.26 ± 2.04. Of the participants, 56.2% came from rural areas and 43.8% from urban areas. Approximately one-quarter (23.4%) were only children in their family. More than half (61.8%) reported holding student leadership positions. Additionally, 68.3% reported nursing was their first-choice major. Detailed demographic information is presented in Table 1.

**Table 1:** Demographic characteristics of nursing students.

**Variables**	**Total (*n* = 1,832)**	**Male (*n* = 332)**	**Female (*n* = 1,500)**	**Statistic**	***P***
**Age** (Mean ± SD)	18.26 ± 2.04	18.64 ± 2.19	18.17 ± 1.99	*t* = 3.61	< .001
**Source of students**, ***n*** **(%)**				χ^2^ = 0.60	0.438
Urban	802 (43.78)	139 (41.87)	663 (44.20)		
Rural	1,030 (56.22)	193 (58.13)	837 (55.80)		
**Only child**, ***n*** **(%)**				χ^2^ = 15.24	< .001
Yes	429 (23.42)	105 (31.63)	324 (21.60)		
No	1,403 (76.58)	227 (68.37)	1,176 (78.40)		
**Monthly household income per capital**, ***n*** **(%)**				χ^2^ = 6.97	0.137
≤ 1000RMB	123 (6.71)	28 (8.43)	95 (6.33)		
1,001–3000RMB	363 (19.81)	58 (17.47)	305 (20.33)		
3,001–5000RMB	519 (28.33)	95 (28.61)	424 (28.27)		
5,001–7000RMB	395 (21.56)	61 (18.37)	334 (22.27)		
>7,000 RMB	432 (23.58)	90 (27.11)	342 (22.80)		
**Student leadership experience**, ***n*** **(%)**				χ^2^ = 0.80	0.373
Yes	1,132 (61.79)	198 (59.64)	934 (62.27)		
No	700 (38.21)	134 (40.36)	566 (37.73)		
**Nursing is the first choice**, ***n*** **(%)**				χ^2^ = 9.55	0.002
Yes	1,251 (68.29)	203 (61.14)	1,048 (69.87)		
No	581 (31.71)	129 (38.86)	452 (30.13)		
**Daily internet use time**, ***n*** **(%)**				χ^2^ = 16.89	0.002
< 1h	148 (8.08)	38 (11.45)	110 (7.33)		
1–3h	799 (43.61)	133 (40.06)	666 (44.40)		
3–5h	616 (33.62)	106 (31.93)	510 (34.00)		
5–7h	162 (8.84)	24 (7.23)	138 (9.20)		
>7h	107 (5.84)	31 (9.34)	76 (5.07)		

*t*, t–test; χ^2^, Chi–square test; SD, standard deviation.

### 3.2 Latent profile analysis of self-efficacy in information security and positive coping levels

**Figure 1:**
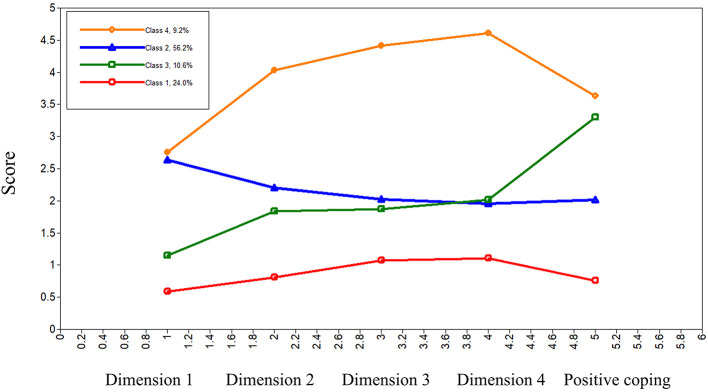
Distribution of latent profile characteristics of self-efficacy in information and positive coping among nursing students.

The fit indices for the latent profile analysis are presented in Table 2. As the number of classes increased from two to five, the AIC, BIC, and aBIC values consistently decreased, indicating an improved model fit. Both the LMRT and the BLRT were statistically significant (*P* < 0.05) for the 2–, 3–, 4–, and 5–class models, suggesting that these tests did not yield a clear optimum among the competing models. Considering the balance between statistical fit, classification quality, parsimony, and interpretability, the four-class model was selected as the optimal solution. The four profiles accounted for 24.0%, 10.6%, 56.2%, and 9.2% of the sample. The entropy value was 0.977, suggesting an excellent classification quality. The score patterns of the four profiles across the four SEIS dimensions are shown in Figure 1.

**Table 2:** The model fitting results of latent profile analysis of SEIS and positive coping (*n* = 1,832).

**Classes**	**AIC**	**BIC**	**aBIC**	**Entropy**	**LMRT**	**BLRT**	**Category probability (%)**
2	20,449.963	20,538.173	20,487.342	0.993	0.000	0.000	90.666/9.334
3	15,994.371	16,115.661	16,045.768	0.988	0.000	0.000	24.236/66.976/8.788
4	14,463.727	14,618.096	14,529.141	0.977	0.000	0.000	10.371/23.963/56.496/9.170
5	13,368.328	13,555.776	13,447.759	0.975	0.0008	0.000	23.854/8.734/52.893/7.751/6.769

Four distinct latent profiles were identified based on the nursing students' scores across the four SEIS dimensions and positive coping. The first profile, labeled the low self-efficacy-negative coping type (24.0% of participants), was characterized by the lowest scores across all dimensions of the SEIS and the lowest levels of positive coping. The second profile, identified as the awareness-dominant type (56.2% of participants), was the largest. Participants in this profile exhibited high judgment capability scores but low scores for security perception, protective behaviors, content control capability, and positive coping. The third profile, termed the low self-efficacy-positive coping type (10.6% of participants), exhibited relatively low scores of the SEIS but comparatively high levels of positive coping. Finally, the fourth profile, labeled the high self-efficacy-positive coping type (9.2% of participants), exhibited the highest levels across all SEIS dimensions and positive coping.

### 3.3 Factors associated with self-efficacy in information security

Prior to the regression analysis, the model assumptions were examined. Multicollinearity was assessed using variance inflation factors (VIF), and all adjusted GVIF values ranged from 1.01 to 1.14, indicating no multicollinearity. Residual normality was evaluated using Q–Q plots and by calculating skewness (0.47) and kurtosis (4.34), which were within acceptable ranges. Homoscedasticity was examined using the Breusch–Pagan test, which indicated heteroscedasticity (BP = 487.33, *P* < 0.001). Therefore, heteroscedasticity-consistent (HC3) robust standard errors were applied in the regression analyses to ensure reliable inference.

Multiple linear regression analysis identified several significant factors associated with the SEIS. Compared with first-grade students, second-grade (β = 0.075, *P* < 0.001), third-grade (β = 0.033, *P* = 0.051), and fourth-grade (β = 0.164, *P* < 0.001) students reported higher SEIS scores. Regarding educational level, all higher categories demonstrated significantly greater scores than the reference group, including associate degree (β = 0.591, *P* < 0.001), undergraduate (β = 0.652, *P* < 0.001), and postgraduate (β = 0.395, *P* < 0.001) students . Students who had not experienced digital victimization had significantly higher SEIS scores than those who had (β = 0.052, *P* < 0.001). In addition, not receiving information security training was associated with lower SEIS scores (β = – 0.074, *P* < 0.001). A lack of clinical internship experience was associated with lower scores (β = – 0.137, *P* < 0.001). Not having completed a nursing informatics course was associated with lower scores (β = −0.056, *P* < 0.001). The overall model was statistically significant [F (13, 1818) = 214.5, *P* < 0.001] and demonstrated a strong explanatory power (adjusted *R*^2^ = 0.603). Given the large sample size (*n* = 1832), the regression estimates were considered robust to minor deviations from normality. The detailed results are presented in Table 3.

**Table 3:** Multivariate linear regression analysis of factors influencing self—efficacy in information security among nursing students.

**Variable**	**Category**	**β**	**Robust SE**	**95% CI**	***P***
Education level	(Technical secondary school)				
	Associate degree	0.591	0.034	0.552–0.629	< 0.001
	Undergraduate	0.652	0.047	0.602–0.701	< 0.001
	Postgraduate	0.395	0.067	0.372–0.418	< 0.001
Grade	(First grade)				
	Second grade	0.075	0.031	0.043–0.107	< 0.001
	Third grade	0.033	0.037	0.000–0.067	0.051
	Fourth grade	0.164	0.075	0.119–0.208	< 0.001
	Fifth grade	−0.014	0.040	−0.033–0.005	0.139
Clinical internship experience	(Yes, currently interning)				
	Yes, internship ended	0.022	0.050	−0.028–0.071	0.394
	No	−0.137	0.044	−0.186−0.088	< 0.001
Completed a course in nursing informatics	(Yes)				
	No	−0.056	0.027	−0.086−0.025	< 0.001
Experience of digital victimization	(Yes)				
	No	0.052	0.025	0.024–0.079	< 0.001
Received information security training	(Yes)				
	No	−0.074	0.028	−0.105−0.042	< 0.001

Reference categories are shown in parentheses.

## 4 Discussion

### 4.1 Heterogeneous patterns of self-efficacy in information security and positive coping among nursing students

This study identified four latent profiles of SEIS and positive coping levels among nursing students in East China. The largest group, the awareness-dominant profile, is consistent with Bandura's self-efficacy theory (Bandura, 1997), which states that efficacy beliefs influence behavior only when supported by adequate skills, confidence, and environmental reinforcement. This suggests that students in the awareness-dominant profile may have a conceptual understanding of information security but lack the behavioral mastery or contextual reinforcement needed to implement security measures effectively (Ussher-Eke, 2023). This discrepancy may be partly attributed to the specific educational context in China, where nursing curricula often prioritize rigorous theoretical instruction and standardized knowledge testing. While this ensures high baseline awareness of regulations, students may have fewer opportunities for autonomous decision-making due to strict hierarchical supervision policies, which may delay the translation of knowledge into behavioral mastery.

In contrast, the high self-efficacy–positive coping profile embodies the optimal combination of cognitive confidence and behavioral readiness. Empirical evidence supports that when self-efficacy is high and paired with positive coping, outcomes such as resilience, psychological wellbeing, and behavioral competence markedly improved (Fitriawan et al., 2023). In digital domains, the presence of coping strategies further moderates the effect of digital demands on burnout and adverse mental health outcomes (Ibrahim et al., 2025). Few students attained this optimal profile, likely because limited mastery experiences, a lack of role models, insufficient encouragement, and heightened stress hinder the simultaneous development of high self-efficacy and positive coping (Bandura, 1997). The small proportion of students in this optimal profile (9.2%) suggests that most students in East China still lack either confidence or coping skills This highlights the need for educational strategies that help students translate their awareness into practical competence.

The “low self-efficacy and negative coping” profile is characterized by behavioral passivity, such as avoidance or helplessness when confronted with security risks. This combination creates vulnerability, and a lack of confidence inhibits proactive measures, perpetuating a cycle of passive insecurity and leaving them susceptible to cyber threats (Siah et al., 2022). Consistent with previous evidence, individuals exhibiting both low self-efficacy and negative coping tendencies have been found to experience poorer psychological adjustment, greater emotional distress, and diminished resilience when facing stressful or threatening situations (Benyon et al., 2010). In contrast, students in the “low self—efficacy—positive coping” profile may exhibit a compensatory form of resilience. Despite low self-efficacy, their use of positive coping strategies may reflect a conscious effort to compensate, driven by positive outcome expectations (Woreta et al., 2025). However, without a robust knowledge and skill base, these efforts may be misdirected or only partially effective. Unsuccessful outcomes could, in turn, lead to frustration and reinforce negative self-perceptions (Zhou et al., 2020).

Furthermore, future studies that incorporate both positive and negative coping strategies may reveal additional latent profiles or substantially alter the composition of the existing profiles. Including negative coping dimensions could lead to the identification of more heterogeneous or mixed coping patterns. For instance, profiles characterized by low self-efficacy but moderate levels of positive coping may be further differentiated according to the presence of negative coping tendencies, thereby uncovering subgroups with distinct psychological vulnerabilities. Such an expanded analytical framework would provide a more nuanced understanding of how positive and negative coping strategies jointly interact with SEIS, ultimately facilitating the development of more precisely targeted educational and psychosocial interventions.

### 4.2 Factors associated with self-efficacy in information security

Multivariate regression analysis identified several factors significantly associated with SEIS in East China. A higher education level was significantly positively associated with the SEIS. This suggests that as nursing students progress to higher academic ranks, they are likely to accumulate more exposure to digital tools, data handling practices, and complex clinical scenarios that boost both knowledge and confidence in secure information behaviors. Students without digital victimization experiences had higher SEIS scores, possibly because they maintained a more positive perception of their ability to manage information security. In the absence of negative experiences, they are less likely to doubt their skills or feel overwhelmed by security challenges, thereby sustaining a higher sense of self-efficacy (Maurya et al., 2023). Moreover, cyber-victimization undermines self-efficacy (Schunk et al., 2022). Such negative experiences may reduce individuals' perceived control and confidence in managing information security, thereby lowering their SEIS. In contrast, several factors were negatively associated with SEIS: the absence of a nursing informatics course, information security training, and clinical internship experience. These findings highlight the importance of formal educational exposure and practical experience in East China. Without coursework in informatics or dedicated training, students may lack structured access to key security concepts, reducing opportunities for mastery, feedback, and situational learning that fosters self-efficacy. Similarly, the lack of clinical internship experience impeded SEIS development. Internships provide real-world contexts in which students manage information systems, encounter workflow vulnerabilities, and practice secure behaviors (le Roux et al., 2024; Park and Jeong, 2021); this exposure is critical for building confidence. However, within the context of nursing education in China, interns often have limited access to hospital information systems, as clinical training primarily focuses on nursing operations rather than on digital data management. Consequently, even during internships, students may lack opportunities to actively engage with security protocols, further restricting the practical reinforcement needed to build SEIS. Therefore, clinical training programs should balance traditional nursing skills with structured opportunities for students to access, manage, and interact with digital health data under supervision. By integrating both formal education and practical, digitally engaged clinical experiences, nursing students can develop not only competence and confidence in secure information practices but also a stronger sense of professional accountability in the evolving e-health landscape.

### 4.3 Educational implications and significance of self-efficacy in information security

These findings highlight the need for differentiated strategies to enhance nursing students' SEIS across the four identified profiles in East China. As digital environments become integral to clinical communication and professional learning, fostering students' confidence, competence, and preparedness to act is essential for maintaining secure and ethical digital practice.

Although the four profiles differ in terms of efficacy and coping tendencies, they collectively reveal a key challenge: translating cognitive awareness into consistent, regulated behavior. Therefore, educational interventions should prioritize strengthening the cognition—behavior linkage in East China. For students with an awareness—dominant profile, educational strategies should integrate experiential modules, such as simulated cyber-risk scenarios, interactive decision-making tasks, and guided feedback. By engaging in self-regulatory processes, these interventions help transform abstract knowledge into practical skills. (Brydges et al., 2015). Peer collaboration and reflective debriefing further reinforce this transition by promoting self-monitoring and sustained behavioral control (Huang et al., 2024). Second, the two low-efficacy profiles share a vulnerability rooted in inadequate mastery experiences, a core determinant in Bandura's self-efficacy theory (Bandura, 1997). Accordingly, stepwise skill-building, scaffolded feedback, and structured success experiences are essential for fostering a sense of control (Huang et al., 2020). Students with negative coping tendencies may require a more supportive learning environment, such as peer-assisted learning and instructor modeling to counter avoidance-based appraisals, consistent with the transactional model of stress and coping (Lazarus and Folkman, 1984; Onieva-Zafra et al., 2020). For students in the “high self-efficacy—positive coping” profile, the educational focus should shift from direct intervention to peer sharing and guidance. These students can serve as models by sharing their experiences with peers through collaborative learning or mentoring activities. Facilitating such peer-led interactions not only reinforces their own sense of competence but also helps other students internalize positive behaviors and enhance their SEIS.

Beyond influencing security-related behaviors, SEIS also strengthens nursing students' awareness of their ethical responsibilities in handling digital information (Park and Jeong, 2021). Students with higher SEIS tend to exhibit greater vigilance, stronger intention to protect privacy, and clearer understanding of the consequences of unsafe digital practices. In summary, SEIS is central to shaping nursing students' engagement with digital practices and perceptions of potential risks. By cultivating students' commitment to privacy and their understanding of behavioral consequences, SEIS promotes more consistent and responsible handling of digital information. This underscores the practical significance of assessing and supporting SEIS in nursing education in East China, as students with higher SEIS are more likely to maintain safe digital practices, thereby enhancing both individual and organizational security.

### 4.4 Limitations and future research directions

This study has several limitations. First, the cross-sectional design limits the causal interpretation of the relationship between the SEIS profiles and positive coping levels. Second, the sample was drawn using convenience sampling from a limited number of nursing institutions in China, which may limit the representativeness of the participants and the generalizability of the findings. Students who chose to participate might differ systematically from those who did not, potentially introducing selection bias. Third, all data were collected through self-reported measures, potentially introducing response bias and common method variance in the results.

Future studies should explore the mechanisms linking SEIS and positive coping levels. Longitudinal or intervention-based designs could clarify causal pathways and assess the effectiveness of training programs targeting these two constructs. Additionally, cross-cultural comparisons may shed light on how sociocultural norms shape the interplay between digital confidence and coping strategies among nursing students. Addressing these areas can help future research better understand how to cultivate information security skills and foster psychologically adaptive healthcare professionals in the digital era.

## Data Availability

The datasets generated and analyzed during the current study are available from the corresponding author on reasonable request. Requests to access the datasets should be directed to Yifang Li, lyf20110801@163.com.
